# Evidence for Inflammation as a Driver of Atrial Fibrillation

**DOI:** 10.3389/fcvm.2020.00062

**Published:** 2020-04-29

**Authors:** Xiaoxu Zhou, Samuel C. Dudley

**Affiliations:** Division of Cardiology, Department of Medicine, the Lillehei Heart Institute, University of Minnesota at Twin Cities, Minneapolis, MN, United States

**Keywords:** atrial fibrillation, driver, inflammatory biomarker, atrial remodeling, anti-inflammatory treatment

## Abstract

Atrial fibrillation (AF) is one of the most common types of arrhythmias and increases cardiovascular morbidity and mortality. Current therapeutic approaches to AF that focus on rhythm control have high recurrence rates and no life prolongation value. While possible explanations include toxicity of current therapies, another likely explanation may be that current therapies do not address fundamental mechanisms of AF initiation and maintenance. Inflammation has been shown to affect signaling pathways that lead to the development of AF. This paper reviews the roles of inflammation in the occurrence, development, and mechanisms of AF and reviews the therapeutic implications of the correlation of inflammation and AF.

## Introduction

Atrial fibrillation (AF) is one of the most common abnormal heart rhythms. AF influences more than 33.5 million people worldwide (1, 2). AF is associated with clinical consequences that reduce the quality of life and increase mortality from cardiovascular disease (3). The onset and maintenance of AF may have different mechanisms, but it is clear that structural and electrical remodeling perpetuate AF. Structural remodeling includes atrial fibrosis, a process closely related to inflammation (4). Inflammatory infiltration has been observed in atria of AF patients (5), and inflammation is known to affect signaling pathways for AF development (4). In this review article, the relationship between inflammation and AF, possible novel mechanistic understandings, and therapeutic approaches arising from this association will be discussed.

## The Relationship Between Inflammation and the Pathogenesis of AF

Inflammation can alter atrial electrophysiology and structure to increase the vulnerability to AF. These two effects are known as atrial electrical and structural remodeling, respectively. AF initiation, by triggers, and maintenance, by a change in substrate, are likely by distinct but overlapping mechanisms.

A major substrate for chronic AF is thought to be atrial fibrosis and the associated slowing and disarray of conduction (6, 7). Evidence for the relationship of AF and fibrosis includes the degree of atrial fibrosis positively associated with AF persistence (8) and the occurrence and recurrence of postoperative AF in patients undergoing open heart surgery (9, 10). The observed changes in atrial structure during AF include atrial dilatation, atrial cardiomyocyte hypertrophy, dedifferentiation, fibrosis, apoptosis, and myolysis (11). Fibrosis is a hallmark of structural remodeling and is an important AF substrate (11). Overexpressing TGFβ1, a profibrotic cytokine, increases atrial fibrosis and vulnerability of AF (12). TNF-α, discussed below, may contribute to AF by activating the TGF-β/Smad2/3 signaling pathway to induce atrial fibrosis (13). In addition, Galectin-3 is thought to act as a marker of fibrosis (14), and elevated levels of circulating galectin-3 predict the prevalence and incidence of AF (15). These examples point out a plausible link between inflammation and AF through structural remodeling.

AF is a hypercoagulable state, and hypercoagulability is associated with systemic inflammation and can promote fibrosis. In adult atrial fibroblasts, thrombin has been shown to cause fibrotic and inflammatory responses. In transgenic mice, enhanced thrombin increased the episodes of AF. In AF goats, decreased thrombin generation reduced AF complexity and AF-related fibrosis. These results suggest that activated coagulation plays a potential role in atrial remodeling (16).

AF electrical remodeling is classically thought to include action potential shortening, reducing electrical connections between cells, and alterations in Ca^2+^ handling. Connexins form gap junctions linking atrial myocytes electrically, and alterations of the distribution and amount of atrial connexin 40 and connexin 43 are associated with inflammation (17). Furthermore, NF-κB may alter the expression of the sodium channel, which is the main channel generating current for conduction (18). Therefore, there are plausible ways in which inflammation may contribute to electrical remodeling and the risk for AF.

## Evidence for an Association Between Local Inflammation and AF

Local inflammatory conditions, including pericarditis and myocarditis, are associated with a high incidence of AF (19). Consistent with local inflammation contributing to the arrhythmia, AF patients have immune cell infiltrates in the atria (20), and activation of leukocytes is increased in patients with perioperative AF (21). This suggests that immune cell infiltration in the atria may be a link between inflammation and AF. For example, AF patients have higher CD45^+^ lymphocytes (22) and CD68^+^ macrophages counts in the atria than do controls.

Suggesting a role for innate immune responses, cardiac MCP-1, a cytokine that can recruit monocytes, dendritic cells and memory T cells, is increased in AF patients (23) and is also associated with circulating fibrosis biomarkers (24). The level of MCP-1-Induced Protein is increased in age-related AF patients compared with the other groups (24). Toll-like receptors (TLRs) are involved in innate immunity, and TLR 2 and 4 have been shown to be potential novel biomarkers for new-onset AF after acute myocardial infarction (25).

The NLRP3 (NACHT, LRR, and PYD domain containing protein 3) inflammasome is an important inflammatory signaling complex and central to innate immunity. The components of NLRP3 inflammasome have been found in both cardiomyocytes (CMs) and cardiac fibroblasts (7). NLRP3 inflammasome activation contributes to formation of caspase-1 to produce the active forms of interleukins-18 (IL-18) and IL-1β, two pro-inflammatory cytokines (26). The activity of NLRP3 inflammasome is elevated in the atrial CMs from paroxysmal and chronic AF patients (27), and expressions of NLRP3, ASC, and active caspase-1 (Casp1-p20) are increased in persistent AF patients (28). In a mouse with constitutive expression of active NLRP3, spontaneous atrial ectopy and inducible AF occur (27). Furthermore, in AF dogs and spontaneous AF mice, the activity of the NLRP3 inflammasome is enhanced in atrium (27). Therefore, the activation of the NLRP3 inflammasome leads to electric and structural remodeling in atrium to contribute to AF (28). Proposed mechanisms linking NLRP3 activation to AF include abnormal sarcoplasmic reticulum Ca^2+^ release (7), action potential shortening, and atrial hypertrophy.

## Evidence of a Relationship Between Systemic Inflammation and AF

Inflammation is a necessary biological process for the defense of organisms against pathogens (29, 30). Nevertheless, uncontrolled inflammation constitutes a positive feedback loop that can lead to numerous unintended diseases (20). There is considerable evidence to support a direct relationship between inflammation and AF (31). For example, new-onset AF has been observed frequently in the critically ill or in patients with sepsis (32, 33). Pneumococcal pneumonia has also been shown to be associated with AF (34).

The systemic inflammatory response after coronary artery bypass grafting is correlated with AF occurrence (35). In the FIBRO-RISK study, biomarkers of inflammation and myocardial fibrosis were validated as predictors for AF recurrence (36). Elevated inflammatory biomarkers levels in serum are correlated with prevalence and prognosis of AF (37, 38). In a prospective pilot study including patients with paroxysmal AF undergoing catheter ablation, patients with up-regulation of inflammatory biomarkers had more frequent early recurrence of AF in the first post-ablation week (39).

The relationship of AF with systemic inflammation is suggested by an increased prevalence in patients with autoimmune diseases. Rheumatoid arthritis (RA) is correlated with the development and maintenance of AF (40–42). In many clinical studies, the prevalence of AF is shown to be increased in RA patients (43). In a collagen-induced arthritis rat model, RA can cause AF and increase AF duration (44), suggesting roles in the initiation and maintenance of the arrhythmia (41).

Other systemic inflammatory conditions are associated with AF. Psoriasis is correlated with increase of AF risk in a nationwide cohort of these patients (45). In large population-based studies and in meta-analyses, psoriatic arthritis patients have a higher AF risk than in the general population (45–47). Furthermore, the psoriasis severity tracks with AF risk, suggesting a cause and effect relationship (45). In inflammatory bowel disease (IBD) patients, P-wave dispersion, a risk factor for AF development, is higher than that in healthy individuals (48). Furthermore, atrial electromechanical delay is higher in active IBD patients (49, 50), again suggesting a cause and effect relationship between the two conditions. In patients with systemic sclerosis and systemic lupus erythematosus, AF and atrial ectopic beats are frequent, and the rate of transient AF occurrence may be 20–30% (51). In ankylosing spondylitis patients, the risk of AF has been shown higher than in the general population in large population-based studies and meta-analyses (52–54). In summary, local and systemic inflammation are associated with AF. Nevertheless, the relationships vary depending on the type of inflammation. This suggests the possibilities that inflammation may be a stronger causative agent in some types of AF than in others and that different types of inflammation may be more or less proarrhythmic.

## Inflammatory Biomarkers are Correlated With AF

Biomarker profiles can predict AF risk and the prognosis after AF ablation (55, 56). Nevertheless, the initiating and sustaining factors for AF may be different, and inflammation has been associated with both. In a study on patients with new onset AF, the early recurrence of AF was related to inflammatory markers, and inflammatory markers were associated with development of permanent AF (57). A list of possible inflammatory factors correlated with AF is presented in (Table 1), and data supporting the associations are discussed below.

**Table 1:** A list of possible inflammatory markers associated with AF.

**Inflammatory factor**	**Function in inflammation**	**Role in AF**	**References**
CRP	A nonspecific inflammatory biomarker	Elevated CRP predicts the incidence of AF, and there is a dose dependence of CRP and AF risk.	(58–67)
MCP-1	A chemokine for monocytes and macrophages	Elevated MCP-1 is associated with AF and circulating fibrosis biomarkers in patients.	(23, 24)
NLRP3 inflammasome	Producing active forms of IL-1β and IL-18	NLRP3 inflammasome activation contributes to atrial electric and structural remodeling to lead to frequent atrial ectopy and reproducible pacing-induced AF.	(7, 26–28)
TNF-α	Inducing inflammation	Higher level of TNF-α is linked to greater risk of AF.	(23, 61, 68–70)
IL-1	Regulating inflammatory responses	IL-1β activation may play a role in pressure overload-induced sustained AF.	(71)
IL-2	Involved in the inflammatory process	IL-2 can cause atrial electrical remodeling and is a predictor of AF after cardioversion.	(72–74)
IL-6	Stimulating inflammatory responses	Increased levels of IL-6 are associated with increased incident AF in patients.	(61, 75–79)
IL-8	Promoting leukocyte migration	Higher IL-8 levels are increased in subjects with longer AF durations or permanent AF.	(5, 80)
IL-18	A proinflammatory cytokine	Increased IL-18 is associated with AF recurrence.	(74)

C reactive protein (CRP), an acute-phase protein whose circulating concentrations rise in response to inflammation, is increased in patients with AF (58). Moreover, there is a dose dependence of CRP and AF risk (59), and elevated CRP predicts the incidence of AF after cardioversion, catheter ablation or cardiac surgery (60–62). The AF-CRP relationship seems to hold for the high sensitivity test. High-sensitivity CRP is associated with AF development and persistence (63), and predicts increased mortality in AF patients (64). Nevertheless, CRP and AF were not correlated when measured before cardioversion (65), and CRP has not been helpful when used to predict postoperative AF (66, 67). These findings indicate that CRP may not be pathogenic in AF and that there may be different relationships between AF and inflammation depending on the inciting cause or longevity of the AF. For example, it is conceivable that inflammation has different relationships to the initiation and maintenance of AF. Alternatively, different types of inflammation may have varying degrees of effect on AF.

Interleukins (ILs) are a group of cytokines involved in the inflammatory response. IL-1 is a key factor regulating innate immune and inflammatory responses. IL-1β is secreted by activated macrophages. There is evidence that IL-1β activation is involved in pressure overload-induced AF (71). IL-2 directly affects T cell activation. IL-2 is associated with shortening of the action potential duration (72), and increased blood levels of IL-2 are correlated with AF risk in the early period after coronary bypass surgery (73). In addition, IL-2 can predict AF after cardioversion (74). IL-6 acts as a pro-inflammatory cytokine and initiates activations of Janus Kinase and Ras-mediated signaling. IL-6 is upstream of CRP and TNF-α production (75, 76). Therefore, it stands to reason from the above discussion that high levels of serum IL-6 are linked to the recurrence of AF after electrical cardioversion and catheter ablation (61). Elevated circulating IL-6 levels have also been correlated with increased incidence of AF (77) and development of AF in postoperative bypass surgery patients (78). Nevertheless, IL-6 does not differ between new-onset AF and chronic AF, suggesting that IL-6 may be better associated with initiation than maintenance of AF (79). IL-8 induces migration of leukocytes and leads to phagocytosis (5). IL-8 levels are increased in the right atrium and coronary sinus in permanent AF patients, compared with paroxysmal AF patients, and elevated IL-8 levels have been shown also in those with longer AF durations (80). After cardioversion, the IL-18 level is elevated in patients with AF recurrence (74, 80). Despite the relationship of some inflammatory cytokines to AF risk, the direct pathogenic role of these cytokines remains to be established.

TNF-α, a cytokine present in systemic inflammation, is associated with fibrosis (68). Higher TNF-α levels are linked to greater risk of AF (61) and with AF presence in the setting of valvular disease (69). The levels of TNF-α are also increased in patients with permanent AF, compared with patients with paroxysmal AF (23). Furthermore, soluble TNF-α predicts exercise-induced AF vulnerability (70). Finally, myeloperoxidase (MPO) produces hypohalous acid in response to infection. Circulated MPO predicts AF recurrence after AF ablation (81).

## Correlation of Inflammatory Cell Populations and AF

White blood cells (WBC) possibly play a role in AF pathogenesis. In the Framingham Heart Study, WBC count was associated with the incidence of AF (82) and with AF recurrence in a recent meta-analysis (83). The neutrophil/lymphocyte ratio (NLR), a routinely available marker of the systemic inflammatory response, predicts AF after bypass surgery (84). The NLR also predicts AF occurrence and recurrence in patients undergoing ablation or surgery (85–87). In a retrospective study in patients with acute AF who were successfully converted to sinus rhythm by amiodarone, NLR was shown to predict AF recurrence (88). Nevertheless, in another prospective cohort study in patients undergoing a successful electrical cardioversion in non-valvular AF, NLR was not useful to predict AF recurrence (89).

In summary, systemic inflammatory diseases are associated with AF, and a worsening inflammatory state is related to a higher AF risk. These relationships suggest a cause and effect conjunction. Given the wide range and systemic nature of the inflammatory diseases associated with AF, the implication is that the immune effect on the heart can be transmitted by blood and that a wide range of systemic inflammatory mediators may trigger AF.

## Inflammation Suppression And AF

Another line of evidence suggesting a relationship between inflammation and AF comes from the antiarrhythmic effects of suppression of inflammation. Possible anti-inflammatory treatments for AF are shown in (Table 2).

**Table 2:** A list of possible anti-inflammatory treatments for AF.

**Anti-inflammatory agents**	**Effect on inflammation**	**Main effect on AF treatment**	**References**
Corticosteroid	Having potent anti-inflammatory effect	Corticosteroid reduces the recurrence of AF following cardiac surgery and ablation.	(90–95)
Colchicine	Having anti-inflammatory effect	Colchicine is associated with decreased rates of postoperative AF and reduces early AF recurrence after catheter ablation.	(74, 96–99)
ACEI and ARB	Inhibiting the renin angiotensin system	Both ARBs and ACEIs reduce AF burden and angiotensin II-induced fibrotic remodeling.	(100–102)
Aldosterone antagonists	Reducing inflammation, oxidative stress, and fibrosis	These agents reduce AF burden and fibrotic pathways.	(103–105)

Corticosteroids have a potent anti-inflammatory effect on reducing recurrence of AF after ablation therapy (90, 91) and post-operative AF after cardiac surgery (91, 92). Moreover, low-dose corticosteroids prevent the recurrence of AF (93). Just as the relationship of inflammatory markers and AF was not uniform, high dose steroids are associated with increase of arrhythmia risk (94) and, in one trial, steroids did not impact the clinical outcomes of AF ablation (95). Since steroids have multiple side effects and a complex effect on AF, they may not be an ideal therapy. Nevertheless, steroids affecting AF is another line of evidence supporting the relationship of AF and inflammation.

Colchicine has an anti-inflammatory effect (96), and short-term use of colchicine has been linked to decreasing the rates of postoperative AF (97) and also reducing early recurrence of AF after catheter ablation (98). In a meta-analysis, colchicine decreases AF after cardiac surgery or radiofrequency ablation (99). This beneficial effect of colchicine on preventing AF has been attributed to the decreased level of CPR and IL-6 (74).

Inflammation is closely related to the activation of the renin angiotensin system (RAS). Both angiotensin receptor blockers (ARBs) and angiotensin-converting enzyme inhibitors (ACEIs) can reduce new-onset AF in patients with hypertension. In a study of 82 patients with paroxysmal AF, an ACEI and an ARB reduced AF burden and inflammation (100). In a mouse model, AF can be inhibited by suppressing angiotensin II-induced fibrotic remodeling (101). Suggesting complexity of the relationship between RAS and AF, the administration of an ACEI and an ARB increased the risk of AF after cardiac bypass in a retrospective study (102). Aldosterone is part of the same RAS system, and aldosterone antagonists might be beneficial for AF via decreasing inflammation, oxidative stress, and fibrosis (103). Spironolactone treatment significantly prevented the alterations of atrial structure and function and reduced fibrotic pathways in a canine model with persistent AF. Spironolactone therapy also reduced the burden of AF in human studies (104). Moreover, mineralocorticoid receptor antagonists attenuate postoperative and heart failure related AF (105), and eplerenone can reduce AF burden (105).

Other treatments suggesting a relationship between AF and inflammation include TNF-α gene ablation preventing structural remodeling of the atrium and decreased vulnerability of AF in response to exercise in exercised mice (70). Moreover, renal denervation can decrease inducibility of AF via reduction of sympathetic RAS activity and inhibition of inflammation and fibrosis in a model of renal impairment (106).

## Summary

AF is a complex disease with a multifactorial etiology. Data reviewed in this article supports the idea that inflammation may be one driver of AF. Mechanisms of inflammation-related AF include inflammation-induced alteration of electrophysiological properties, initiation of early and late afterdepolarizations, remodeling of cardiac structure, and enhanced fibrosis. These inflammatory factors induce the occurrence of ectopic activity and re-entry which contribute to the initiation and maintenance of AF. Inflammatory diseases are associated with AF, AF is characterized by increased inflammatory markers, and anti-inflammatory treatments decrease AF risk. Both systemic and cardiac localized inflammation are associated with AF risk.

Nevertheless, inflammation is multifaceted, and different types of inflammation appear to affect AF risk differently. Moreover, inflammation seems to have variable effects on different types and durations of AF. Therefore, there is much left to sort out about the relationship between AF and inflammation.

## Prospects and Limitations

Current AF therapies are limited by high rates of recurrence and complications. Generally, they fail to address the underlying pathology and exacerbate ion channel dysregulation and structural inhomogeneities that are linked to disease progression. Along with the unchanged mortality rates with a rhythm management strategy, this situation implies that we have a good deal more room for better therapeutic options. Moreover, it suggests that the current strategies may not be addressing fully the underlying pathophysiological drivers of AF. One such driver appears to be inflammation.

While a clear causal link between AF and inflammation has yet to be determined, anti-inflammatory therapeutic strategies may be a next logical step in AF care, one not burdened by proarrhythmic risk.

## Author Contributions

XZ wrote the content of the manuscript. SD provided critical review.

## Conflict of Interest

The authors declare that the research was conducted in the absence of any commercial or financial relationships that could be construed as a potential conflict of interest.
